# Some Byeways of Practice

**Published:** 1896-08-15

**Authors:** 


### Some Byeways of Practice.

A solicitor, travelling by a Southern railway with a medical friend the other day, suddenly sprang upon him the question whether or not a new order of medical practitioners had recently come into existence. The man of law had, he said, come into contact with medical men who, on their door-plates and in other ways, described themselves as "physicians and surgeons." The doctor was rather taken aback for the moment; but, after a little reflection, he gave it as his opinion that a practitioner who describes himself as "physician and surgeon" generally does so because, in point of actual fact, he is neither a "physician" nor a "surgeon," but a hybrid, having the defects of both, but the virtues of neither.

This opinion, we fancy, will meet with pretty general endorsement among self-respecting practitioners. It is no doubt true that many persons are licentiates both in medicine and surgery; and in that sense they are perhaps entitled to call themselves "physicians and surgeons" if they please. But inasmuch as the word "physician" has a generally accepted significance of very long standing, and the word "surgeon" is in exactly the same case, it is better for practitioners to keep these words to the uses which are universally understood. For a licentiate in medicine and surgery to call himself a "physician and surgeon" is disingenuous, and unworthy of a gentleman. It is also manifestly misleading, because if it misleads a man of the status of a solicitor, it is quite clear that it is likely to mislead men and women of any other class. This we insist should not be done. If a man is a general practitioner he can put Dr. on his door-plate, or Mr. ——, Surgeon, and then everybody knows what is meant. It ought to be the aim of the doctor, as of every other person, to be as straightforwardand truthful with the public, as he is in private, and to avoid all misleading pretences as he would avoid lying or theft.

Under the same category of fraudulent misrepresentation may be placed the conduct of the man in the suburbs of populous towns who puts in large letters on a flaring window pane the announcement that he is a "specialist" in diseases of the throat, or eye, or in midwifery, or vaccination, or what-not. As a plain matter of fact he is nothing of the kind. He is a general practitioner, who may or may not have had opportunities of seeing a little special practice, and who is clearly entitled to take what fair, but no unfair, advantage he can from his special knowledge. But to announce to all and sundry that he is an actual specialist in a particular disease such as has been named, when he knows, and every other medical man knows, he is a general practitioner, and that of a very humble class, is dishonest and shameful, and would be felt to be so if any proper sense of shame were still left. We sympathise very sincerely with those of our brethren who find themselves grievously put to it to make headway in the struggle for survival. But we cannot sympathise with misrepresentation and fraud.

A very sorry byeway, not to say "dirty lane" of practice is followed by the fully qualified medical man who unblushingly announces in the most public way he can that he gives "advice and medicine for sixpence." There only needs the consideration of one fact to demonstrate that this kind of practice is and must be purely fraudulent. That fact is this, that the chemist whose shop is in the very same neighbourhood cannot afford to give medicine alone for sixpence. If it be said the mention of the sixpence is merely a "decoy" used to get the patient into the surgery, and that if once he is in the doctor must be a very stupid person who cannot make at least two shillings out of him, then all we can reply is that the system is not merely a fraud, it is also a blackguardism. We cannot but express the wish that medical men, before they finally decide to enter upon the *rôle* of the "sixpenny dispenser" would take counsel of their better selves, and refuse, even for bread, to play the part of deliberate cheats in the world. There is much need of amendment in many of the practices that prevail among us, and we cannot but think that if the British Medical Association or one of the "Defence and Protection societies" would take the matter seriously up a great deal of good might gradually be done.

